# Citronellal Exerts Its Antifungal Activity by Targeting Ergosterol Biosynthesis in *Penicillium digitatum*

**DOI:** 10.3390/jof7060432

**Published:** 2021-05-29

**Authors:** Qiuli OuYang, Yangmei Liu, Okwong Reymick Oketch, Miaoling Zhang, Xingfeng Shao, Nengguo Tao

**Affiliations:** 1 School of Chemical Engineering, Xiangtan University, Xiangtan 411105, China; qiuliouyang@xtu.edu.cn (Q.O.); 201921001766@smail.xtu.edu.cn (Y.L.); reymick.oketch@mak.ac.ug (O.R.O.); mling_z@xtu.edu.cn (M.Z.); 2 Department of Food Science and Engineering, Ningbo University, Ningbo 315211, China; shaoxingfeng@nbu.edu.cn

**Keywords:** citrus, pathogen, ergosterol, ERG genes, cell membrane integrity

## Abstract

Ergosterol (ERG) is a potential target for the development of antifungal agents against *Penicillium digitatum*, the pathogen of green mold in citrus fruits. This study examined the mechanism by which citronellal, a typical terpenoid of *Cymbopogon nardus* essential oil, acts on ergosterol to exhibit its antifungal activity against *P. digitatum*. We previously reported that citronellal inhibited the growth of *P. digitatum* with minimum inhibitory concentration (MIC) and minimum fungicidal concentration (MFC) of 1.36 and 2.72 mg/mL, respectively. In citronellal-treated cells, the membrane integrity and ergosterol contents significantly decreased, whereas lanosterol, which serves as a precursor for ergosterol biosynthesis, massively accumulated. Addition of 150 mg/L of exogenous ergosterol decreased the inhibitory rate of citronellal, restoring the ergosterol content and hence the membrane structure to normal levels, and triggered expression of nearly all ERG genes. Based on our findings, we deduce that citronellal damages the cell membrane integrity of *P. digitatum* by down-regulating the ERG genes responsible for conversion of lanosterol to ergosterol, the key downregulated gene being *ERG3*, due to the observed accumulation of ergosta-7,22-dienol.

## 1. Introduction

Green mold, caused by the pathogen *Penicillium digitatum*, is the major postharvest disease of citrus during harvesting and postharvest handling processes [1]. Currently, the disease is mainly being managed by use of chemical fungicides, but the extensive use of such fungicides has complicated the process through emergence of drug-resistant strains [2]. As a consequence of the increased emergence of resistance to these chemical fungicides, exacerbating by their negative environmental impacts, alternative drug targets including use of plant essential oils have been explored.

Essential oils (EOs) are considered safer, highly active, and with low possibility to induce drug resistance [3,4,5,6], thus together with some of their volatile compounds, they have gradually been applied to prevent postharvest diseases such as grey mold and green mold [7,8,9]. Citronellal, one of the main constituents of *Cymbopogon nardus* essential oil, has a strong inhibitory effect against fungi [10,11]. Previously, citronellal was reported to be effective in inhibiting the growth of three pathogenic *Penicillium* strains including *P. digitatum*, *P. italicum*, and *P. ulaiense*, with the inhibition zone ranging from 31 to 43 mm^2^ [12]. Recently however, Morcia et al. [13] and Wang et al. [14] confirmed that citronellal not only inhibits the growth of *Penicillium* species but also of *Fusarium* and *Aspergillus* species. Our previous study demonstrated that citronellal visibly inhibited the germination of *P. digitatum* spores at concentrations higher than 0.17 mg/mL and delayed the green mold decay of navel orange in a dose-dependent manner [15]. In addition, citronellal inhibited the mycelial growth of *P. digitatum*, with minimum inhibitory concentration (MIC) and minimum fungicidal concentration (MFC) of 1.36 and 2.72 mg/mL respectively. Combined with cinnamaldehyde, citronellal obviously reduced the green mold incidence in Satsuma mandarin citrus fruit and did not impair the fruit quality [16]. Citronellal may therefore be used as a novel natural antifungal alternative for the effective preservation of citrus fruits.

Existing literature suggests that cell membrane is the action target of citronellal against fungi. For example, citronellal inhibited the growth of *Candida albicans* by affecting membrane integrity and arresting cell cycle [17]. In addition, Singh et al. (2016) [18] reported that citronellal interferes with membrane homeostasis by increasing the hypersensitivity of fungi to membrane-perturbing agents, reducing ergosterol levels, and diminishing glucose-induced H^+^ extrusion. Ergosterol, an integral component of the cell membrane that plays a key role in maintaining the integrity and fluidity of the membrane structure, is a key target of common chemical fungicides (such as imazalil and prochloraz) against *P. digitatum* [2,19]. Indeed, a number of studies have shown that essential oils such as citral, eugenol, thymol, carvacrol, and perillaldehyde can significantly reduce the ergosterol content in the cell membranes of *P. digitatum*, *Aspergillus* species, and *Candida* species, causing abnormal cell membrane function [20,21,22,23,24,25,26]. Although we previously reported that citronellal inhibited *P. digitatum* by damaging the cell membrane integrity thereby leading to leakage of intracellular constituents [15,16], the actual mechanism by which citronellal causes damage to the cell membrane of *P. digitatum* is still unknown. This study was therefore designed (i) to evaluate the effect of citronellal on the plasma membrane integrity of *P. digitatum*, (ii) to determine its effect on lanosterol and ergosterol contents and on sterol composition, and (iii) to analyze its effect on gene expression levels in ergosterol biosynthesis of *P. digitatum*.

## 2. Materials and Methods

### 2.1. Pathogens

*Penicillium digitatum* used in this study was isolated from infected Satsuma mandarin (*Citrus unshiu* Marc. cv. Miyagawa Wase), identified by morphological and molecular biology methods, and preserved at Xiangtan University [27]. This strain was incubated on potato dextrose agar (PDA) at 25 ± 2 °C and their spore concentrations adjusted to 5 × 10^6^ spores/mL using a hemocytometer.

### 2.2. Fruit

Mature mandarin fruits (*Citrus reticulata* Blanco) were harvested on 21 December 2019 from an orchard in Luxi, Hunan, China. Healthy fruits of uniform size and without scars were selected for the experiments.

### 2.3. Chemicals

Citronellal (96%) was purchased from Aladdin Chemical Reagent Co., Ltd. (Shanghai, China), solutions prepared by dissolving the requisite amount in Tween-80 (0.05%, *v*:*v*) and topped up to the final volumes using distilled water. All the chemicals were of analytic grade.

### 2.4. In Vivo Experiments of Citronellal

Artificial inoculation of fruits with *P. digitatum* and their subsequent treatment with citronellal were conducted using the method described by OuYang et al. [16] with minor modifications. All fresh fruits were surface-sterilized by immersing in 2% sodium hypochlorite solution (*v*/*v*) for 2 min, then washed with distilled water, wounded (depth of 3 mm and width of 3 mm) with a sterile needle, inoculated with 20 μL of *P. digitatum* spore suspension (10^5^ spores/mL), and left to air-dry. The inoculated fruits were then immersed in in wax amended with citronellal at 1 × and 5 × MFC for 30 s. Fruits with wax and pathogen inoculation were used as control. Ten mandarin fruits constituted a single replicate, and each treatment was performed in triplicate. The incidence rate of disease (measured by counting the number of green mold-infected wounds) was calculated from the formula:

(1) $$Disease\;incidence=\frac{Number\;of\;rotten\;wounds}{Total\;number\;of\;wounds}\times 100$$

The diameter (mm) of lesion was measured using a G102-123-101 caliper (Shanghai Measuring and Counting Tools Co., Ltd., Shanghai, China).

### 2.5. Additional Exogenous Ergosterol Assay

Effects of addition of external ergosterol on the antifungal activity of citronellal against mycelial growth of *P. digitatum* were tested in vitro by agar dilution method [15] using final citronellal concentrations of 0, 1/4 MIC, 1/2 MIC, MIC, and 2 MIC. Additional exogenous ergosterol assay was performed according to the method by Nóbrega et al. [28] to compare the antifungal activity of citronellal in the absence and presence of ergosterol at concentrations of 50, 150, and 250 mg/L. Each treatment was performed in triplicate and the culture plates incubated at 25 ± 2 °C for 4 d.

### 2.6. Plasma Membrane Integrity

The plasma membrane integrity of *P. digitatum* cells treated with citronellal (0 or 1/2 MIC) were analyzed by propidium iodide (PI) staining coupled with an ECLIPSE TS100 microscope (Nikon, Japan) and the fluorescence value determined by an F97 PRO fluorescence spectrophotometer (Lengguang Technology, Shanghai, China) as described in OuYang et al. [29]. Each of these experiments was repeated with addition of 150 mg/L of exogenous ergosterol.

### 2.7. Determination of Total Lanosterol and Ergosterol Contents

Total lanosterol and ergosterol contents of *P. digitatum* cells citronellal (1/2 MIC concentration) for 0, 30, and 60 min were determined by high performance liquid chromatography (HPLC) as we previous described [25] with minor modifications. The 2-day-old mycelia were dried in a vacuum freeze drier for 4 h. About 0.008 g of the dry mycelia were homogenized with liquid nitrogen and suspended in 4 mL of freshly prepared 25% (*w*/*v*) NaOH and 8 mL of absolute ethanol, then saponified at 85–90 °C for 2 h. The mixtures were extracted thrice with petroleum ether and washed twice in saturated NaCl solution. The upper organic layer was vacuum concentrated and each residue adjusted to 10 mL using ethanol. The detected wavelengths for lanosterol and ergosterol were set at 210 nm and 282 nm, respectively, calculating lanosterol and ergosterol contents from the standard calibration curve using lanosterol and ergosterol as standards, respectively. Control samples were not treated with citronellal, and the experiments were repeated with addition of 150 mg/L exogenous ergosterol.

### 2.8. Determination of Sterols Composition by Gas Chromatography-Mass Spectrometry (GC-MS)

The composition of sterols in *P. digitatum* cells treated with 1/2 MIC citronellal for 0, 30, and 60 min were determined by GC-MS method [25]. The dry mycelia were saponified with a freshly prepared mixture of NaOH (4 mL, 25% *w*/*v*) and absolute ethanol (8 mL) at 80 °C for 1 h. The mixtures were subsequently extracted in petroleum ether and washed using saturated NaCl solution and then the upper organic layer vacuum concentrated. Each concentrated sample was completely dissolved in 950 μL of methyltertbutylether and then the silylation reagent mixture (50 μL, N-methyl-N-trimethylsilyltrifluoroacetamide and N-trimethylsilylimidazole, *v*/*v*, 9:1) added and left at room temperature for at least 30 min to complete the silylation reaction followed by GC-MS analysis. Control samples were not treated with citronellal. Similar experiments were performed with addition of exogenous ergosterol (150 mg/L) to the *P. digitatum* culture medium.

The analytical GC was carried out on a ShimadzuQP2010 plus gas chromatograph (Shimadzu, Kyoto, Japan) equipped with flame ionization detector (FID). A non-polar cross-linked fused-silica capillary column, HP-5MS (30 m × 0.25 mm × 0.25 μm; Agilent, Santa Clara, CA, USA), was used. The oven temperature was held at 50 °C for 1 min, programmed at a rate of 50 °C/min to 260 °C, then increased to 300 °C at a rate of 4 °C/min where it remained for 10 min. The carrier gas was helium (1.3 mL/min). The injector temperature was 250 °C, detector temperature 310 °C and the volume injected was 2 μL. MS analysis was carried out on the same chromatograph equipped with a Shimadzu QP 2010 GC/MS system, ionization voltage 70 eV, ion source temperature 200 °C, mass range *m*/*z* 50–700, scanning interval 0.5 s and scanning speed 1000 amu/s. The sterol TMS ethers were identified by comparison with commercial references, the NIST™ database, or data from literature [25,30].

### 2.9. Real-Time Fluorescence Quantitative PCR (RTFQ-PCR) Analysis

RNA of *P. digitatum* treated with citronellal (0 or 1/2 MIC concentration) for 0, 30, and 60 min was extracted using the Trizol reagent (Invitrogen, Carlsbad, CA, USA) following the manufacturer’s instructions. The expression levels of the *ERG1*, *ERG2*, *ERG3*, *ERG4*, *ERG5*, *ERG6*, *ERG7*, *ERG9*, *ERG11 ERG24*, *ERG25*, *ERG26*, and *ERG27* genes were examined using RTFQ-PCR. RTFQ-PCR was performed through a BIO-RAD CFX Connect Thermal Cycler using FastStart Universal SYBR Green Master (Roche, Switzerland) with the following programs: 95 °C for 10 min followed by 40 cycles at 95 °C for 15 s, and at 60 °C for 1 min. Actin gene was used as internal reference, and the samples were quantified by 2^−^^△△CT^ method [31]. All primer pairs for expression assays are listed in Table 1. The experiments were repeated with addition of exogenous ergosterol (150 mg/L) to the fungal culture medium.

### 2.10. Statistical Analysis

All data were expressed as their mean ± SD by measuring three independent replicates and analyzed using one-way ANOVA followed by Duncan’s test to test the significance of differences between means obtained among the treatments at the 5% level of significance using SPSS statistical software package release 16.0 (SPSS Inc., Chicago, IL, USA).

## 3. Results

### 3.1. In Vivo Experiments of Citronellal

**Figure 1:**
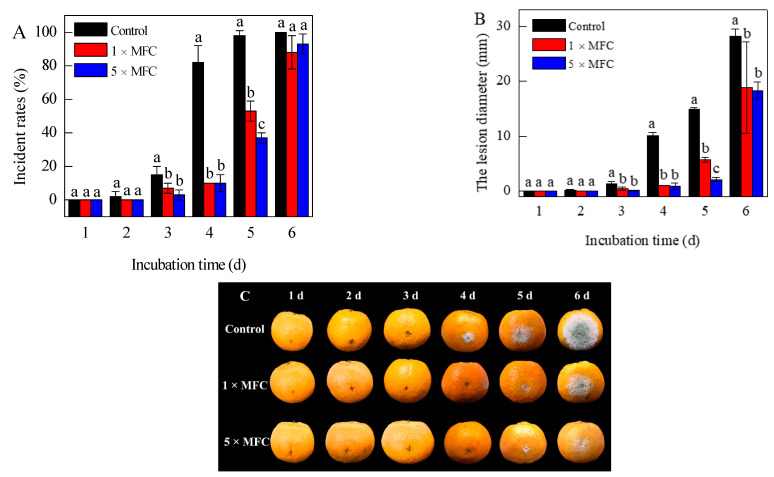
Disease incidence (**A**), the citrus lesion diameter (**B**) and the disease progression (**C**) in inoculated citrus fruits treated with citronellal (0 ×, 1 ×, and 5 ×  MFC) during storage at 25  ±  2  °C for 6 d and 85–90% RH. The data presented are the means of pooled data. Error bars indicate the SDs of the means (*n* = 3).

Citronellal (1 × and 5 × MFC) effectively reduced the green mold decays of citrus fruits by the fifth day of inoculation, being able to alleviate the disease progression in inoculated fruits as shown in Figure 1. By day 4 of incubation with *P. digitatum*, the incidence rate in wax-treated fruits was 82%, a value much higher than the 10% observed in citrus fruits coated with citronellal (1 × and 5 × MFC) (Figure 1A). The average lesion diameter also reduced from 10.08 ± 0.67 mm observed in the control group to 1.07 ± 0.03 and 0.91 ± 0.58 mm in the 1 × and 5 × MFC citronellal treated groups, respectively (Figure 1B,C).

### 3.2. Additional Exogenous Ergosterol Assay

Results of the inhibitory effect of addition of exogenous ergosterol on the antifungal activity of citronellal against *P. digitatum* are presented in Table 1. The MIC and MFC values of citronellal against *P. digitatum* were 1.36 and 2.72 mg/mL, respectively. After 4 days of treatment, the antifungal rate of citronellal (0.78 mg/mL = 1/2 MIC concentration) against *P. digitatum* was 59.60 ± 9.18%. This rate significantly reduced to 42.38 ± 7.16% following addition of external ergosterol (150 mg/L) (Table 2). This result showed that addition of 150 mg/L ergosterol could improve the tolerance of *P. digitatum* to citronellal.

### 3.3. Effects of Citronellal on the Plasma Membrane Integrity

**Figure 2:**
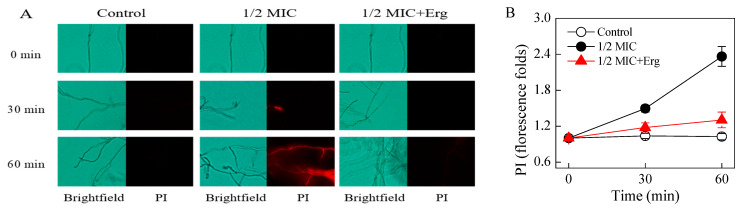
Effects of citronellal on the plasma membrane integrity of *P. digitatum* (**A**) PI staining images of *P. digitatum* mycelia treated with citronellal for 0, 30, and 60 min; (**B**) the fluorescence fold changes of the PI staining). The data presented are the means of pooled data. Error bars indicate the SDs of the means (*n* = 3).

Mycelia treated with 1/2 MIC citronellal showed stronger red fluorescence after 30 min of treatment compared to the controls (Figure 2A). After 60 min of treatment, the fluorescence intensity of the 1/2 MIC group, as determined by the fluorescence spectrophotometer, was 2.36 times higher than that of the control group (Figure 2B), indicating that citronellal had greatly damaged the cell membrane. Within the same 30 min duration of time, mycelia treated with 1/2 MIC citronellal plus externally added ergosterol showed no red fluorescence. Their fluorescence intensity after 60 min of treatment as measured by the fluorescence spectrophotometer was only 1.28 times higher than that of the control group (Figure 2B), representing a significantly lower damage to the membrane compared to that caused in the absence of externally added ergosterol.

### 3.4. Lanosterol and Ergosterol Contents

**Figure 3:**
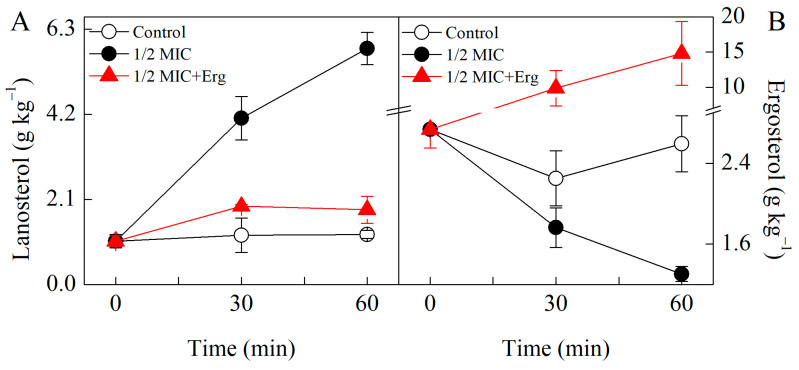
HPLC analysis of lanosterol (**A**) and ergosterol (**B**) contents in control samples, 1/2 MIC citronellal treated samples, and 1/2 MIC citronellal + Erg treated sample. The data presented are the means of pooled data. Error bars indicate the SDs of the means (*n* = 3).

The lanosterol content in citronellal treated *P. digitatum* samples was markedly higher than those of the control groups (Figure 3). Following 30 min of treatment with citronellal, lanosterol content in the test group was 3.4 times higher than in control group. External addition of ergosterol resulted into much lower lanosterol values in the test group, being only 1.6 times higher than those of the corresponding control group (Figure 3A).

Citronellal treatment reduced the ergosterol content of *P. digitatum* but 1/2MIC+Erg treatment induced a higher ergosterol content compared to the control sample (Figure 3B). After 30 min of treatment with citronellal, the ergosterol content of the test group was 21.7% lower than that of the control group. However, treatment with exogenous ergosterol for the same duration of time, raised ergosterol content to a value 4.4 times higher than in control group (*p* < 0.05) (Figure 3B).

### 3.5. Analysis of Sterols Composition by GC-MS

GC-MS results showed that addition of both citronellal and ergosterol could significantly change the steroid components in *P. digitatum* (Table 3). Ergosterol, lanosterol and squalene were continuously detected in both test and control groups all through the experiment. Whereas both ergosta-5,7,22,24(28)-tetraenol and ergosta-7,22-dienol were detected in the control groups, with the former being detected at 30 min and the latter at 60 min, ergosta-7,22-dienol was also detected in the citronellal treated samples at both 30 and 60 min and in the citronellal + ergosterol group at 30 min of treatment. A new compound, eburicol, was detected only in the citronellal treated samples at 30 and 60 min but not in the control group or citronellal + ergosterol group.

### 3.6. Effect of Citronellal on Gene Expression Levels in Ergosterol Biosynthesis

**Figure 4:**
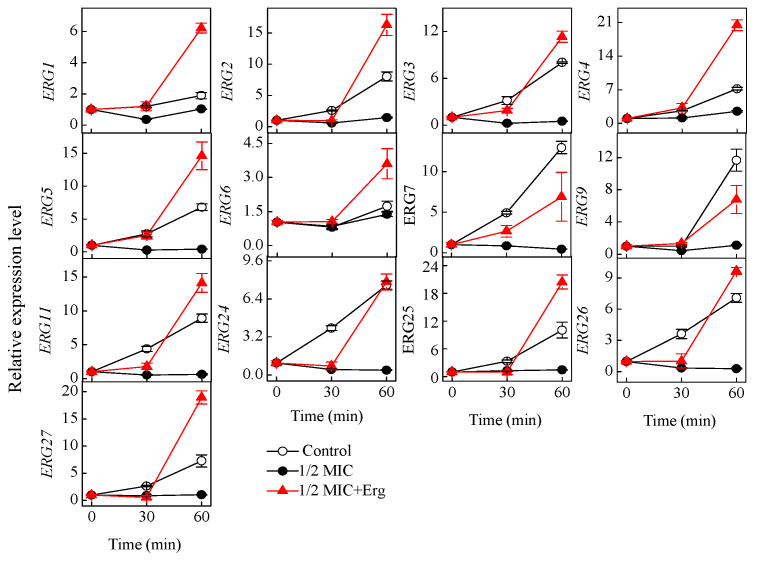
Changes in the expression of ergosterol biosynthesis genes of *P. digitatum* mycelia treated by control, 1/2 MIC citronellal treated samples, and 1/2 MIC citronellal + Erg treated samples for 0, 30, and 60 min. The data presented are the means of pooled data. Error bars indicate the SDs of the means (*n* = 3).

To explore the effect of citronellal on ergosterol biosynthesis of *P. digitatum* at the molecular level, the transcription levels of 13 genes involved in the biosynthesis of ergosterol was investigated. After citronellal treatment, the expression levels of all the 13 ERG genes in the test group (except *ERG6* gene whose level did not change) were repressed (Figure 4).

*P. digitatum* showed different responses at the molecular level when the citronellal-treated cells were supplemented with ergosterol, with the *ERG1*, *ERG3*, *ERG5,* and *ERG7* genes showing increased expression levels at 30 min, and by 60 min, the expression levels of nearly all genes (except *ERG7*, *ERG9,* and *ERG24*) in the test groups being significantly higher than in the control groups (*p* < 0.05) (Figure 4).

## 4. Discussion

Ergosterol is the most abundant and main sterol component of fungal membranes [32], involved in regulation of membrane permeability and fluidity; regulation, activity, and distribution of integral membrane proteins; and control of the cell cycle [33]. It is thus key to the structural integrity of the cell membranes and, as such, the main target of the majority of the available antifungals [32,33], which interfere with its function either through inhibition of various steps in ergosterol biosynthesis or by complexing directly with membrane ergosterol [24]. Numerous essential oils and their components such as *Curcuma longa* L. essential oil, citral, perillaldehyde, and thymol have an impact on the cell membrane integrity by targeting ergosterol metabolism [22,23,24,25,26].

Citronellal is a promising antifungal compound with good inhibitory effects on *P. digitatum* both in vitro and in vivo. Our results show that treatment of *P. digitatum* with citronellal led to a significant inhibition of ergosterol biosynthesis and damage to the cell membrane. This observation corresponds to that reported by Singh et al. [18] in which citronellal had adverse effects on cell membrane integrity and ergosterol levels of *C. albicans*. Addition of 150 mg/L ergosterol to the growth medium greatly enhanced the tolerance of *P. digitatum* to citronellal, restoring the cell membrane integrity and ergosterol content to normal levels. This again is consistent with reports that the presence of exogenous ergosterol in growth medium promotes increased survival of some fungi under test agents stress [24,28,34,35]. Abe and Hiraki [36] and Liu et al. [35] also reported about the restoration of ergosterol content and cell membrane integrity to normal levels following exogenous ergosterol treatment of *Saccharomyces cerevisiae*. These findings confirm a direct correlation between ergosterol levels and cell membrane integrity and allude to the possibility that the antifungal effect of citronellal on *P. digitatum* is attributed to its inhibition of ergosterol biosynthesis.

Ergosterol biosynthesis is tightly regulated by ERG genes [37]. Our previous study showed that citral, another terpene aldehyde, decreased the expression levels of genes *ERG3*, *ERG5*, *ERG6*, and *ERG11* and acted on the ergosterol biosynthesis of *P. digitatum* by targeting the *ERG11* gene [25], and Xu et al. [38] also noted that linalool (a terpene alcohol) downregulated the expression of genes in the ergosterol biosynthesis pathway. We have found similar results in *P. digitatum* in which the expression of most of the ERG genes in citronellal-treated samples was markedly lower (*p* < 0.05) than in the control samples during the entire experimental time. In this pathway, catalyzing C14-demethylation of lanosterol by *ERG11* is critical for ergosterol biosynthesis. Obviously, citronellal induced the downregulated of *ERG11* gene and then led the exclusive accumulation of lanosterol, indicating that the ergosterol synthesis was hindered. Meanwhile, the side chain of ergosterol biosynthesis was activated, inducing the production of eburicol in citronellal-treated samples. These results are consistent with those reported by Ottilie et al. [39] that posaconazole, a *TcCyp51* inhibitor, can increase the accumulation of both lanosterol and eburicol. It should also be noted that the loss of the *ERG3* led to the typical fungal sterol ergosterol and ergosta-5,7,22,24(28)-tetraenol depletion, activating another side chain of ergosterol biosynthesis hence inducing the generation of ergosta-7,22-dienol [40]. This implies that the action site of citronellal against *P. digitatum* might be between the *ERG11* and *ERG3*.

Exogenous ergosterol is significant in improving the synthetic ability of ergosterol in *S. cerevisiae* and *C. glabrata* [34,35]. In this study, ergosterol content markedly increased after 30 min of treatment with 1/2 MIC + Erg. This might have resulted from the coordinated uptake and biosynthesis of the sterols [34]. We also discovered that the expression of genes involved in ergosterol biosynthesis in *P. digitatum* was altered after supplementation of exogenous ergosterol in the citronellal treatment samples. In the citronellal-treated cells supplemented with ergosterol, genes *ERG1*, *ERG3*, *ERG5*, and *ERG7* responded quickly and in just 30 min had already been upregulated more than those in citronellal-treated samples. By 60 min of treatment, all the genes in the test samples supplemented with ergosterol had been upregulated more than those in only citronellal treatment. This corresponds to the result by Liu et al. [35] that ergosterol supplementation triggered the upregulation of genes encoding ergosterol biosynthesis, leading to increase in sterol levels. Furthermore, upregulation of *ERG3* and *ERG5* genes led to a decrease in lanosterol content to the normal value and a decrease in eburicol (a branch chain product) content to the detection limit after 30 min of treatment with exogenous ergosterol. This implies that *ERG3* is the vital regulatory gene.

In summary, the present study has demonstrated that the suppression of ergosterol biosynthesis in *P. digitatum* cells by citronellal treatment occurs between *ERG11* and *ERG3* genes and that gene *ERG3* in *P. digitatum* may be the key regulatory site gene in response to citronellal treatment. These findings provide new insights into the antifungal mechanism of citronellal.

## Figures and Tables

**Table 1:** Primer pair sequences designed for ERG genes in *P. digitatum* using RTFQ-PCR.

Genes	Primer (5′-3′)	Length (bp)
*ERG9*	TCTTTGTTGAGGCCGGGTTT ACTTGGGGCGTTTCAACAGA	51
*ERG1*	ATCCCCGATAACCTGCCTCT CCCTTGACGCCTCCATTCTT	52
*ERG7*	GCGCTGGCGATTGGTCGATG CAGGCCCAGTTTCCGGGCTCC	219
*ERG11*	GCGGAATCAAGAGGGACGAT GCCCTAGCACACACTTCAGA	150
*ERG24*	AGAGCTTCACAGTTCCAGCC CGATGCCTCGCTGACAAATG	155
*ERG25*	ATCGAAAGCTTCCTACGGGG GCGCATCAATAGGCTGAGGA	80
*ERG26*	ACCAGACCCCCTGCATCTAT TTGGGATCCGTGCTCTAGGA	162
*ERG27*	TTTCGATCTGCTGCCGTCTT GCGCGCTTCGAGTTGTAAAT	91
*ERG6*	CGCGTGATGCCGCCTTCAAC TGAGCCTTGCGGGCCTCACG	184
*ERG3*	CAGGCCATGGCCGCAATGCC GGTGCAGGCCACGGTGGATCC	190
*ERG5*	TCTCGCCATTGGCGGATGCG GGCCAACAATGGCGCCCTTG	240
*ERG4*	GCTGGAACCGCTACTTCCTT AGACAAACAGGTAGGCGACG	51
*ERG2*	ACATCTTCGACCCGGAACAC TTGGGACCGACTTTCTGCTC	121
*actin183*	TGCGCTGAACCGAACTGCCG TCGGGAGCCTCGAAGCGCTC	183

**Table 2:** Effects of citronellal and exogenous ergosterol on the growth of *P. digitatum*.

Treatments	Inhibitory Rate (%)			
	1 d	2 d	3 d	4 d
1/4 MIC	40.48 ± 4.12 c	19.42 ± 3.36 d	16.67 ± 4.12 e	14.57 ± 1.99 f
1/4 MIC+50 mg/LErg	42.86 ± 7.14 c	23.30 ± 3.67 d	16.67 ± 2.38 f	21.19 ± 1.15 e
1/4 MIC+150 mg/LErg	40.48 ± 4.12 c	5.82 ± 3.36 f	3.17 ± 2.75 g	5.30 ± 3.03 g
1/4 MIC+250 mg/LErg	50.00 ± 7.14 c	16.50 ± 3.36 e	19.84 ± 5.99 e	15.23 ± 5.00 f
1/2 MIC	66.67 ± 8.25 b	76.70 ± 2.91 c	68.25 ± 5.99 c	59.60 ± 9.18 c
1/2 MIC+50 mg/LErg	66.67 ± 8.25 b	80.58 ± 6.06 b	65.08 ± 4.96 c	57.62 ± 8.96 c
1/2 MIC+150 mg/LErg	52.38 ± 4.12 bc	76.70 ± 2.91 c	50.79 ± 7.27 d	42.38 ± 7.16 d
1/2 MIC+250 mg/LErg	52.38 ± 8.25 b	82.52 ± 5.04 b	74.60 ± 7.65 c	65.56 ± 11.30 c
MIC	100.00 ± 0.00a	100.00 ± 0.00a	95.24 ± 0.00 b	90.73 ± 1.15 b
MIC+50 mg/LErg	100.00 ± 0.00 a	100.00 ± 0.00 a	95.24 ± 0.00 b	85.43 ± 14.91 b
MIC+150 mg/LErg	100.00 ± 0.00 a	100.00 ± 0.00 a	95.24 ± 0.00 b	93.38 ± 1.15 b
MIC+250 mg/LErg	100.00 ± 0.00 a	100.00 ± 0.00 a	95.24 ± 0.00 b	93.38 ± 1.15 b
MFC	100.00 ± 0.00 a	100.00 ± 0.00 a	100.00 ± 0.00 a	100.00 ± 0.00 a
2 MIC+50 mg/LErg	100.00 ± 0.00 a	100.00 ± 0.00 a	100.00 ± 0.00 a	100.00 ± 0.00 a
2 MIC+150 mg/LErg	100.00 ± 0.00 a	100.00 ± 0.00 a	100.00 ± 0.00 a	100.00 ± 0.00 a
2 MIC+250 mg/LErg	100.00 ± 0.00 a	100.00 ± 0.00 a	100.00 ± 0.00 a	100.00 ± 0.00 a

Note: ‘Erg’ represents ergosterol. Data presented are the means ± standard error of pooled data (*n* = 3). Different lowercase letters indicate significant differences of columns at each time point according to Duncan’s multiple range test (*p* < 0.05).

**Table 3:** Effects of citronellal on the sterols composition of *P. digitatum* by GC-MS.

Substances	Control			1/2 MIC		1/2 MIC + Erg	
	Time (min)						
	0	30	60	30	60	30	60
squalene	+	+	+	+	+	+	+
ergosterol	+	+	+	+	+	+	+
ergosta-5,7,22,24(28)-tetraenol	−	+	−	−	−	−	−
ergosta-7,22-dienol	−	−	+	+	+	+	−
lanosterol	+	+	+	+	+	+	+
eburicol	−	−	−	+	+	-	−

Note: ‘+’: detected; ‘−’: not detected.

## Data Availability

The datasets generated during and/or analyzed during the current study are available from the corresponding author upon reasonable request.
